# Effect of wild bitter gourd treatment on inflammatory responses in BALB/c mice with sepsis

**DOI:** 10.7603/s40681-014-0017-y

**Published:** 2014-08-27

**Authors:** Shin-You Ciou, Cheng-Chin Hsu, Yueh-Hsiung Kuo, Che-Yi Chao

**Affiliations:** 1 Department of Nutrition, China Medical University, Taichung, Taiwan; 2 Department of Nutrition, Chung Shan Medical University, Taichung, Taiwan; 3 Department of Chinese Pharmaceutical Sciences and Chinese Medicine Resources, China Medical University, Taichung, Taiwan; 4 Department of Biotechnology, Asia University, Taichung, Taiwan; 5 Department of Health and Nutrition Biotechnology, Asia University, Taichung, Taiwan

**Keywords:** Wild bitter gourd;, Sepsis,, Inflammation;, LPSP;, PARs

## Abstract

Background/Introduction: Wild bitter gourd (Momordica charantia L. var. abbreviate Seringe) common vegetable in Asia, is used in traditional medicine to treat various diseases, including inflammation. Extant literature indicates that wild bitter gourds have components that activate PPARα and PPARγ. This research probed influence of adding wild bitter gourd to diets on inflammation responses in mice with sepsis.

Purpose: This study evaluated influence of eating wild bitter gourd on inflammation responses in mice with sepsis.

Methods: We injected intraperitoneal LPS to induce sepsis. Male BALB/c mice were divided normal, sepsis, positive control, and three experimental groups. The latter ate diets with low (1%), moderate (2%), and high (10%) ratios of wild bitter gourd lyophilized powder. Before mice were sacrificed, with the exception of the normal group, intraperitoneal injection of LPS induced sepsis in each group; positive control group was injected with LPS after PDTC.

Results: This experiment revealed weights in groups with added wild bitter gourd starkly lower than those of the remaining groups. Blood lipids (TG, cholesterol, and NEFA) were also lower in comparison to the sepsis group, and blood glucose concentrations recovered and approached normal levels. Blood biochemistry values related to inflammation reactions indicated GOT, GPT, C-RP, and NO concentrations of groups with wild bitter gourd added all lower than that of the sepsis group. Secretion levels of the spleen pro-inflammatory cytokines IL-1, IL-6, and TNF-α tallied significantly lower in comparison to the sepsis group, whereas secretion levels of IL-10 anti-inflammatory cytokine increased. Expression level of proteins NF-κB, iNOS, and COX-2 were inhibited significantly.

Conclusion: Wild bitter gourd in diets promoted lipid metabolism, improved low blood glucose in sepsis, and attenuated inflammatory stress. These findings suggested that this plant food might provide medical benefits for certain persons.

## 1. Introduction

Wild bitter gourd (*Momordica charantia* L. var. *abbreviate* Seringe), a *Momordica charantia* variety, is consumed as both a vegetable and folk medicine in Taiwan [1,2]. Most noticeable pharmacological property of wild bitter gourd (WBG) is the hypoglycemic activity, not only used widely in herbal medicine but also demonstrated in rodent models [3] and Type 2 diabetic patients [4]. WBG is known to contain numerous components: e.g., alkaloids, steroidal glucosides, phenolics [5], lysophosphatidylcholines [6], conjugated linolenic acid isomers [7], cucurbitane-type triterpenoids [8,9]. WBG is considered more potent in disease prevention than bitter gourd, yet little is known about WBG’s biological and physiological characteristics. Its anti-inflammation property is thus linked to categorization as a “cooling food” in traditional/folk medicine [10]. Precise mechanisms and well-controlled clinical trials of its anti-inflammation effects have not been completely delineated [11].

Inflammation reactions are closely related to progression of and tissue damage caused by numerous diseases, making research on anti-inflammatory effects crucial. Sepsis pathogens notably relate to lipopolysaccharides (LPS) (a.k.a. endotoxins) released by Gram-negative bacteria cell walls. Body cells are stimulated by microbial components. Through production of cytokines and tumor necrosis factor-α (TNF-α), a series of complements, white blood cells, and vascular endothelial cells are activated. Numerous microorganisms produce hypervirulent exotoxins that directly damage human tissue [12]. Inflammation process involves activation of macrophages, inciting expression of cyclooxygenase-2 (COX-2), and leading synthesis of the inflammatory mediator prostaglandin E_2_ (PGE_2_). Prostaglandins produced primarily by macrophages are critical pro-inflammatory mediators [13]. If the formation of inflammatory mediators is reduced, discomfort caused by inflammation is alleviated [14]; this is the purpose of anti-inflammatory drugs.

Peroxisome proliferator-activated receptors (PPARs) are regarded as therapeutic targets for cardiovascular disease: activation of these not only directly regulates genes of vascular and inflammatory cells involved in atherosclerosis, but also indirectly promotes glucose utilization and serum lipid profiles [14]. Prior research in our laboratory pointed to bitter gourd components that activate PPARα and PPARγ [14]. Various bitter gourds (green, white, pearl, wild) on the market in Taiwan were compared by *in vitro* tests to find wild variety with greatest anti-inflammatory capacity. In addition, anti-inflammatory capacities of different sections of this gourd (fruit, seeds, leaves, stems, flowers) were contrasted. The fruit section most effectively inhibited inflammatory mediators PGE_2_ and nitric oxide (NO) [9-11]; we probed anti-inflammatory effects of wild bitter gourd fruit in *in vivo* animal inflammation models (sepsis). We noted influence of bitter melon on animal models with acute septicemia while examining possible mechanisms of action and effect.

## 2. Materials and methods

### 2.1. Preparation of test samples

We homogenized fresh wild bitter gourd (whole fruits including seeds) into a pulp. After using gauze for filtering, we freeze-dried the residue and ground it into powder. For the basic components of the feed, we referenced the AIN-76 formula and made appropriate adjustments, adding various ratios of wild bitter gourd lyophilized powder and dietary fiber. We adjusted dietary fiber content for groups to identical ratios. We added sterile water to shape prepared powder feed into pellets (4 g for each pellet), refrigerating these at -20 °C.

### 2.2. Animal treatment and grouping

Our 72 six-week-old male BALB/c mice (body weight approximately 16-19 g) were purchased from the National Laboratory Animal Center. They were first fed a rodent chow diet for one week of adaptation. When their body weights averaged approximately 22 g, we randomly divided them based on their body weights. Mice were fed for a total of four weeks during the experiment. Feed intake levels during experimentation period were recorded daily, body weight recorded weekly. Each mouse was fed 4 g/d test feed. Excess supply of drinking water was used to allow free intake.

This study used the endotoxin model to induce acute septicemia reactions. Before mouse sacrifice, other than the normal (N) group, each group received intraperitoneal injections of LPS. The positive control (P) group received intravenous injections of anti-inflammatory drug pyrrolidinedithiocarbamic acid ammonium salt (PDTC) 1 hour before the LPS injection. The normal control group was injected with normal saline. Each group fasted overnight (12 hrs) following LPS injections; decapitation was used to sacrifice them. After packaging blood and obtaining organs, these were cryopreserved in refrigerators at -80°C for later analysis. Research accorded with internationally accepted principles for laboratory animal use and care, as reviewed and approved by the Institutional Animal Care and Use Committee Guidelines of China Medical University (IACUC, protocol No: 97-26-N).

Six experimental groups were involved, names and symbols for each indicated below:

1. N: Normal group (i.p. normal saline), fed the chow diet.
2. S: Sepsis group (i.p. LPS, 15 mg/kg BW), fed the chow diet.
3. L: Sepsis group with low-dose (1%) wild bitter gourd lyophilized powder added, fed feed including 1% wild bitter gourd lyophilized powder.
4. M: Sepsis group with moderate-dose (2%) wild bitter gourd lyophilized powder added, fed feed including 2% wild bitter gourd lyophilized powder.
5. H: Sepsis group with high-dose (10%) wild bitter gourd lyophilized powder added, fed feed including 10% wild bitter gourd lyophilized powder.
6. P: Positive control group (i.p. PDTC, 50 mg/kg BW), fed the chow diet.

### 2.3. Blood preparation and biochemical assay

After four weeks’ treatment, mice were fasted overnight (twelve hours) and decapitated. Blood was centrifuged at 1700 X g at 4°C for 30 min to separate serum gauged by enzymatic methods, using commercial kits (RANDOX, Amtrim, UK) for TG, cholesterol, glucose and non-esterified fatty acid (NEFA), these biochemical analysis as previously described.^1^ Serum inflammatory mediator included NO, glutamate oxaloacetate transaminase (GOT), glutamate pyruvate transaminase (GPT) and C-reactive protein (C-RP), with concentration analyzed by clinical test kits (Roche Cobas Mira plus, Germany).

### 2.4. Measurement of splenocyte cytokine levels by ELISA

Splenocyte cytokine levels were determined by commercially available enzyme-linked immunosorbent assay (ELISA) kit (Bio-source International Inc., Camarillo, CA) according to manufacturer’s instructions. Cytokines TNF-α, IL-1β, IL-6 and IL-10 were determined by a standard curve, concentrations were expressed as μg/mL.

### 2.5. Western blot analysis

Liver tissues were homogenized in lysis buffer (0.6% NP-40, 150 mM NaCl, 10 mM HEPES (pH 7.9), 1 mM EDTA, and 0.5 mM PMSF) at 4°C. Fifty microgrammes of protein subjected to 10 % SDS-PAGE was transferred to polyvinylidene fluoride (PVDF) membrane (NEN Life Science, Boston, MA), blot immunodetected with enhanced chemiluminescence (ECL) Western blot kit (Amersham International, Amersham, UK) in which goat anti-mouse COX-2, nuclear factor-kappaB (NF-κB), inducible nitric oxide synthase (iNOS) and β-actin antiserum served as primary antibody (Millipore, MA) and goat anti-rabbit IgG-biotinylated species-specific whole antibody (Calbiochem) was used as secondary antibody. Blots were quantified by relative intensity compared to control with Kodak Molecular Imaging Software (Version 4.0.5, Eastman Kodak Company, Rochester, NY), represented in relative intensities.

### 2.6. Statistical analysis

Our experimental results indicate mean ± SD. After confirming normal distribution, we employed one-way ANOVA and Duncan’s multiple range test to identify differences between independent sample groups, using SPSS 13.0 software (Chicago, IL) for statistical analysis.

**Table 1:** Initial body weight, final body weight, and food intake for mice.

Group	N	Body weight		Food intake
		initial BW(g)	final BW(g)	(g/day)
**N**	12	22.6±2.0^a^	25.8±1.7^b^	3.8±0.2^a^
**S**	12	23.0±1.5^a^	27.0±1.3^a^	3.8±0.3^a^
**L**	12	23.3±1.0^a^	23.5±0.9^c^	3.9±0.3^a^
**M**	12	22.8±1.3^a^	22.8±0.5^c^	3.9±0.3^a^
**H**	12	23.6±1.3^a^	19.8±1.5^d^	3.9±0.6^a^
**P**	12	23.0±0.7^a^	25.2±0.5^a^	3.8±0.2^a^

Values not sharing letters in the same vertical column differ significantly from one another by one-way ANOVA and Duncan’s Multiple Range Test (*p* < 0.05) among six groups.

## 3. Results and Discussion

### 3.1. Effects of wild bitter gourd on body weight change and lipid metabolism

Table 1 shows changes in body weight and intake level for each group: body weights of Groups L, M, and H significantly lower than those of Groups N, S, and P. Group H had most significant weight-loss effect. Organ lesion caused by sepsis spawns metabolic abnormalities for blood lipids (TG, NEFA, and cholesterol) within the body. This is significantly reduced after adding wild bitter gourd, trending toward a dose response. Table 2 shows that the blood glucose abnormalities of Group S followed low blood glucose situations. This may be caused by multiple organ failure (MOF) following sepsis and is considered a complication thereof [18]. Wild bitter gourd is known to activate PPARα, thus moderating lipid metabolism [14,19]; its active components, such as conjugated linoleic acid (CLA), are speculated to activate PPARα, thereby facilitating β-oxidation for fatty acids and maintaining constancy of lipid metabolism within cells [20,21]. PPARα also regulates ketogenesis within the body, thereby moderating metabolism and balance of lipids [21,22].

Earlier studies confirm wild bitter gourd as containing PPARα and PPARγ activators [1] that facilitate lipid metabolism, reduce blood lipids, and cause anti-inflammatory activity [2]. This subsequently reduces fat accumulation, resulting in weight-loss effect. Besides reducing blood glucose concentrations, PPARγ activation reduces inflammation reactions. Molecular mechanisms may entail inhibition of cytokines secreted by monocytes following PPARγ activation [14]. This interferes with transmission of messages for NF-κB, signal transducers and activators of transcription (STAT), activator protein-1 (AP-1), etc., inhibiting expression of inflammatory genes like IL-1, IL-2, IL-6, IL-8, TNF-α, and metalloproteinase (MMPs) [23]. PPAR agonists currently treat atherosclerosis; they activate both receptors simultaneously and are regarded as having greatest potential. Whether significant reduction in body weight of mice fed 10% wild bitter gourd wreaks negative physiological effects merits further research.

**Table 2:** Serum triglyceride, cholesterol, NEFA, and glucose concentrations in mice after four weeks of test diet.

Group	**N**	Triglyceride (mg/dL)	Cholesterol (mg/dL)	NEFA (mmol/L)	Glucose (mg/dL)
**N**	12	186.8±11.7^a^	194.7±20.3^c^	0.64±0.03^d^	102.6±4.8^a^
**S**	12	224.2±10.9^bc^	227.1±36.0^a^	0.95±0.05^a^	50.1±5.2^c^
**L**	12	196.0±6.6^c^	222.9±15.5^b^	0.92±0.01^a^	50.6±4.9^c^
**M**	12	197.3±23.9^c^	222.1±24.2^a^	0.85±0.01^a^	72.7±16.4^b^
**H**	12	188.7±21.4^d^	207.1±13.8^c^	0.83±0.01^b^	64.5±2.1^b^
**P**	12	186.4±7.6^a^	202.9±22.2^b^	0.79±0.01^c^	101.4±9.4^a^

Values not sharing letters in the same vertical column differ significantly from one another by one-way ANOVA and Duncan’s Multiple Range Test (*p* < 0.05) among six groups.

### 3.2. Effects of wild bitter gourd on inflammation response and organ damage

**Fig.1:**
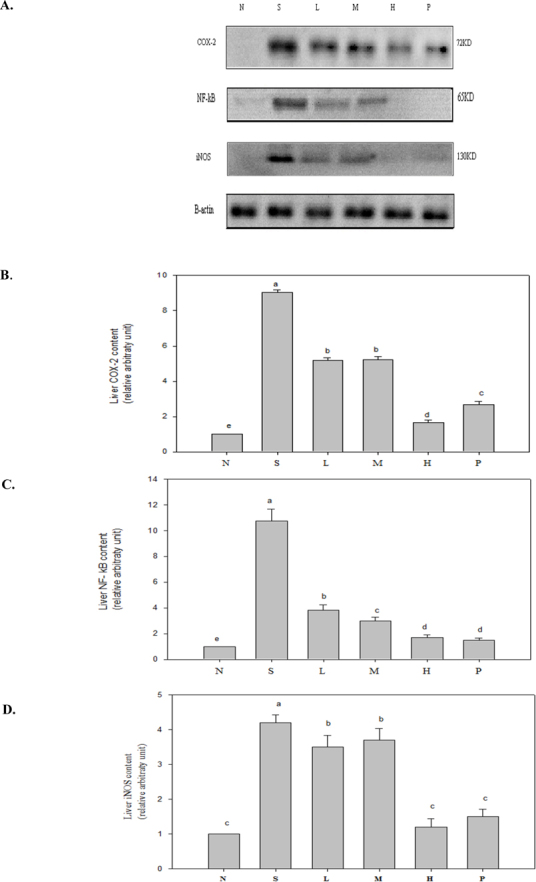
COX-2, NF-κB, and iNOS inflammatory protein expression in six groups of mice after four-week test diet, values mean ± SD of each group. Values not sharing a superscript (a-e) differ significantly by one-way ANOVA and Duncan’s Multiple Range Test (*p* < 0.05) among groups. One representative immunoblot from three independent experiments is shown.

Figure 1 shows expression levels of proteins COX-2, iNOS, and NF-κB (phosphorylated form) increasing significantly within the body during the inflammation reaction process, thus elevating concentrations of downstream inflammatory mediators like PGE2 and NO (data not shown). Table 4 plots pro-inflammatory cytokine (TNF-α, IL-1β, and IL-6) concentrations in spleens of mice with sepsis as definitely higher than those in Group N. These ratios dropped sharply after wild bitter gourd was added to diets. Secretions of anti-inflammatory cytokine IL-10 increased, presenting dose response trends. Serum inflammatory mediators like NO, AST, ALT, and C-RP, all fell significantly in mice after four weeks of test feeds containing various doses of wild bitter gourd, consequently reducing damage to organs and tissue. Group H had most significant results, falling to values equal to those of Group N (Table 3). When organs are damaged, AST (GOT) and ALT (GPT) within cells are released into serum. Extant literature indicates that after sepsis occurs, cell necrosis increases during the septic process, releasing more liver cell enzymes. AST and ALT concentrations in blood rise [24], causing inflammation response accompanied by severe organ damage [25].

LPS is currently known to increase transcriptional effects of iNOS mRNA by activating NF-κB [26]. Two hours following acute attacks of sepsis, NO is produced excessively within kidneys, resulting in low blood pressure and tachycardia [27]. Extant literature indicates that activation of PPARγ can inhibit formation of inflammatory mediators like TNF-α, IL-1β, and IL-6 [28]. PPARγ also reduces iNOS activity by inhibiting transcription factor NF-κB, thence alleviating inflammation [29]. This study indicates 10% wild bitter gourd diet significantly inhibiting expression of iNOS protein, reducing formation of inflammatory mediator NO. Likewise, adding wild bitter gourd to diets can reduce formation of inflammatory mediator PGE2 by reducing expression of COX-2 protein. This reduces production of pro-inflammatory cytokines and other substances. Anti-inflammatory cytokine IL-10 also rises significantly, achieving anti-inflammatory effects. Extant literature indicates butanol-soluble fraction of its placenta extract strongly suppressing LPS-induced TNF-α yield in RAW264.7 cells. Anti-inflammatory components were identified as 1-α-linolenoyl-lysophosphatidylcholine (LPC), 2-α-linolenoyl-LPC, 1-lynoleoyl-LPC, and 2-linoleoyl-LPC [6].

In Groups L, M, and H, since wild bitter gourd contains substances capable of activating PPARγ, severity of septic inflammation reaction was retarded, thus reducing organ damage. Extant literature indicates liver vascular sinus accumulating formation of substantial amounts of platelets, red blood cells, white blood cells, and microthrombosis during endotoxic shock. This causes expansion and obstruction to the vascular sinus, thereby damaging liver function [30]. Acute septicemia causes substantial increases in vascular permeability and rapid heart rate. The ensuing insufficient return of blood to organs affects heart weight and blood volume. This study also indicates that, regarding weights of organs (liver, kidneys, and spleen), Group H proves able to reduce organomegaly (data not shown) compared with Group S. These will help design human studies necessary to prove efficacy of bitter gourd against clinical sepsis.

## 4. Conclusions

Wild bitter gourd in diets facilitates lipid metabolism, reducing blood lipid concentration and body weight. Group H shows most significant results. Adding wild bitter gourd to diets of sepsis-induced mice reduced expression of proteins COX-2, iNOS, and NF-κB, all associated with inflammation. It reduced secretions of pro-inflammatory cytokines and other substances, hence reducing organ damage. As discussed, this study indicates wild bitter gourd lyophilized powder retarding negative effects caused by LPS-induced sepsis using. Its numerous health benefits include reducing blood lipids, improving blood glucose, and combating inflammation. In the future, with active ingredients purified, isolated, and identified, this common crop can be used clinically as nutritional supplement to improve acute endotoxin-induced septicemia.

**Table 3:** Serum inflammatory mediator concentrations in mice after four weeks of test diet.

Group	n	NO (μM)	AST(GOT) (unit/L)	ALT(GPT) (unit/L)	C-RP (ng/mL)
**N**	12	23.8±1.6^c^	27.1±2.4^c^	31.3±2.1^a^	167.0±18.1^b^
**S**	12	39.3±5.1^a^	48.9±3.8^a^	46.6±2.5^b^	227.0±20.0^a^
**L**	12	33.5±2.7^b^	43.1±2.3^b^	40.2±3.0^c^	211.5±10.4^a^
**M**	12	25.7±1.6^b^	40.5±2.6^b^	36.3±2.1^d^	220.9±19.9^a^
**H**	12	24.7±1.5^c^	25.0±2.3^c^	27.4±2.3^a^	181.4±16.7^b^
**P**	12	25.0±3.5^c^	16.6±3.1^d^	19.6±2.4^e^	188.5±17.9^b^

Values not sharing letters in the same vertical column differ significantly from one another by one-way ANOVA and Duncan’s Multiple Range Test (*p* < 0.05) among six groups.

**Table 4:** Spleen level of inflammatory cytokines concentrations in mice after four weeks of test diet.

Group	n	TNF-α (μg/mL)	IL-1β (μg/mL)	IL-6 (μg/mL)	IL-10 (μg/mL)
**N**	12	16.68±0.20^bc^	18.70±0.77^c^	17.64±0.33^d^	20.40±0.33^d^
**S**	12	20.65±0.43^a^	20.78±0.78^b^	23.68±1.26^a^	22.38±0.48^c^
**L**	12	15.72±1.25^d^	18.55±0.78^c^	21.20±0.91^c^	22.48±0.18^c^
**M**	12	17.53±0.36^b^	21.46±0.34^ab^	22.71±1.80^ab^	23.54±0.25^a^
**H**	12	14.37±0.93^e^	16.38±1.76^d^	21.93±0.37^bc^	23.42±0.32^ab^
**P**	12	16.57±0.45^cd^	22.09±0.13^a^	18.71±0.56^d^	23.05±0.31^b^

Values not sharing letters in the same vertical column differ significantly from one another by one-way ANOVA and Duncan’s Multiple Range Test (*p*< 0.05) among six groups.

### Abbreviations

WBG, Wild bitter gourd; LPS, Lipopolysaccharides; COX-2, Cyclooxygenase-2; TNF-α, Tumor necrosis factor-α; PGE2, Prostaglandin E2; PPARs, Peroxisome proliferator-activated receptors; NO, Nitric oxide; PDTC, Pyrrolidinedithiocarbamic acid ammonium salt; NEFA, Non-esterified fatty acid; GOT, Glutamate oxaloacetate transaminase; C-RP, C-reactive protein; GPT, Glutamate pyruvate transaminase; PVDF, Polyvinylidene fluoride; ECL, Enhanced chemiluminescence; NF-κB, Nuclear factor-kappaB; iNOS, Inducible nitric oxide synthase; MOF, Multiple organ failure; CLA, Conjugated linoleic acid; STAT, Signal transducers and activators of transcription; LPC, Lysophosphatidylcholine; AP-1, Activator protein-1; MMPs, Metalloproteinase.

### Acknowledgements

We thank Dr. Zhi-Hong Wang for animal technical supports. This work was supported by research grants (NSC 97-2320- B-468-001-MY3 and NSC 102-2320-B-468-003) from the National Science Council, Taipei, Taiwan and CMU under the Aim for Top University Plan of the Ministry of Education, Taiwan, and the Department of Health Clinical Trial and Research Center of Excellent (DOH102-TD-C-111-004), Taiwan.

### Declaration of Interest:

Authors declare no conflicts of interest for this work.
